# Lac-Phe elicits anxiolytic-like effects associated with monoaminergic signaling in mice

**DOI:** 10.1038/s41398-026-04106-2

**Published:** 2026-05-29

**Authors:** Shiho Suzuki, Kanon Chiba, Hanako Kanzaki, Hisako Takahashi, Yukiko Yamazaki, Nozomi Takahashi, Miho Naoi, Ryoji Kise, Asuka Inoue, Kei Tsuzuki, Yoshiya Seto, Takahiro J. Nakamura, Kentaro Kaneko

**Affiliations:** 1 Department of Agricultural Chemistry, School of Agriculture, Meiji University, 1-1-1, Higashimita, Tama-ku, Kawasaki-shi, Kanagawa Japan; 2 Laboratory of Animal Physiology, School of Agriculture, Meiji University, 1-1-1, Higashimita, Tama-ku, Kawasaki-shi, Kanagawa Japan; 3 Graduate School of Pharmaceutical Sciences, Kyoto University, 46-29 Yoshida-Shimo-Adachi-cho, Sakyo-ku, Kyoto, Japan; 4 Graduate School of Pharmaceutical Sciences, Tohoku University, 6-3, Aoba, Aramaki, Aoba-ku, Sendai, Japan

**Keywords:** Depression, Physiology

## Abstract

Anxiety disorders are a growing public health concern in modern societies burdened with chronic stress and diminishing work motivation, imposing a substantial disease burden across all age groups. Lactoyl-phenylalanine (Lac-Phe), an endogenous metabolite produced during exercise and found in fermented foods, has recently gained attention for its metabolic regulatory functions, yet its potential anxiolytic effects remain unclear. In this study, we report that Lac-Phe administration elicits anxiolytic-like behavior in mice. Oral administration of Lac-Phe significantly increased open-arm exploration in the elevated plus maze (EPM), indicating anxiolytic-like activity. Similar behavioral effects were observed following intraperitoneal and intracerebroventricular administration in the EPM and the novelty-suppressed feeding test. Pharmacological blockade experiments indicated that dopamine D1 and serotonin 5-HT1A receptor signaling contribute to Lac-Phe-induced anxiolytic-like effects. Consistently, intraperitoneal administration of Lac-Phe increased dopamine tissue content in the hypothalamus and striatum and reduced hippocampal *IL-1β* and *IL-6* mRNA expression. Together, these results suggest that Lac-Phe exerts anxiolytic-like effects, potentially involving monoaminergic signaling in the brain, and support further investigation of Lac-Phe as a food-derived metabolite in the context of stress- and anxiety-related outcomes.

## Introduction

Chronic exposure to stress contributes to the high prevalence of anxiety disorders across diverse populations [1]. This reduces motivation and productivity and contributes to the overall prevalence of disease. Stress refers to a state of mental and physical tension and affects specific brain regions, impairing emotional regulation and resulting in behavioral abnormalities. Anxiety disorders, including generalized anxiety disorder, social anxiety disorder, and separation anxiety disorder [2], arise from dysregulation of common neural circuits.

Among these, the dopaminergic reward system is central, as its modulation under stress is critical for selecting appropriate coping strategies [3–6]. Dysregulation of this pathway has been implicated in maladaptive stress responses, and dopamine D1 receptors are important as they mediate stress-induced synaptic plasticity in the medial prefrontal cortex [7]. Dopamine receptors are widely distributed throughout the central nervous system, where they regulate locomotion, cognition, and emotion. Among them, the dopamine D1 receptor subtype mediates acute stress-induced dendritic growth in excitatory neurons of the medial prefrontal cortex.

In parallel, the serotonergic system contributes to the pathophysiology of anxiety. Hyperactivity of serotonergic signaling is associated with the development of anxiety disorders [8], and 5-HT1A receptors are particularly relevant to anxiolytic and antidepressant responses [9, 10]. Presynaptic 5-HT1A autoreceptors in the raphe nuclei inhibit serotonergic firing, whereas postsynaptic 5-HT1A receptors in limbic and cortical regions attenuate neuronal activity. These findings highlight the complementary roles of dopaminergic and serotonergic systems in regulating stress and emotion.

Despite the involvement of these monoaminergic systems, conventional anxiolytics and antidepressants that primarily target them often show insufficient efficacy and are associated with side effects and dependence [11]. Anxiety and depression arise from complex interactions among genetic factors, environmental influences, and individual differences in neural circuits [12], making it difficult for a single pharmacological approach to address all symptoms. These limitations underscore the need to identify novel compounds that can modulate dopaminergic and serotonergic activity through alternative mechanisms.

Lactoyl-phenylalanine (Lac-Phe) is a metabolite consisting of lactate and phenylalanine linked via an amide bond. Li et al. identified Lac-Phe as an exercise-inducible metabolite that contributes to appetite suppression and weight reduction [13], suggesting its role in metabolic regulation. However, the authors primarily addressed metabolic outcomes and reported limited efficacy following oral administration; thus, its potential influence on emotional behaviors remains unclear. The hypothalamus, a key target of Lac-Phe, regulates both appetite and emotional behaviors [14]. This suggests that Lac-Phe affects metabolism and emotion. Interestingly, Lac-Phe is found in fermented foods, such as Parmesan cheese and soy sauce; even some lactic acid bacteria can produce Lac-Phe during fermentation [15].

Given that Lac-Phe is endogenously produced during exercise, has a well-established role in metabolism, and is present in certain fermented foods, we hypothesized that exogenous Lac-Phe administration might modulate anxiety-related behavior. In this study, we investigated the effects of Lac-Phe on anxiety-related behavior in mice using the elevated plus maze (EPM) and novelty-suppressed feeding test (NSFT) and sought to elucidate the mechanisms underlying these effects, including after oral administration.

## Materials and methods

### Materials

N-lactoylphenylalanine (Lac-Phe, (*S*)-*N*-(2-Hydroxy-1-oxopropyl)-L-phenylalanine) was purchased from Sigma-Aldrich (SMB01375). Lactic acid (L-(+)-Lactic acid; Sigma-Aldrich L1750) and L-phenylalanine (L-Phenylalanine; Sigma-Aldrich P2126), used for comparative studies, were obtained from Sigma-Aldrich. Lipopolysaccharide (LPS; from *Escherichia coli* O111:B4; Sigma-Aldrich L4391) was administered intraperitoneally at a dose of 0.8 mg/kg to induce a depression-like state.

SCH23390 hydrochloride (SCH23390; R&D Systems 0925), a highly potent and selective dopamine D1-like receptor antagonist, and WAY100135 dihydrochloride (WAY100135; Tocris Bioscience 1253), a potent and selective 5-HT1A receptor antagonist, were purchased from Bio-Techne (Minneapolis, USA). Based on previously reported protocols, SCH23390 was administered at 1 μg/mouse (i.c.v.), and WAY100135 was administered at 10 mg/kg (i.p.) [16–18].

### Animals and housing conditions

Male C57BL/6 mice were purchased from the Japan SLC (Shizuoka, Japan). Seven-week-old mice were used for the behavioral tests. All mice were maintained on a 12-h light/dark cycle (lights on 7 a.m.–7 p.m.) in a temperature-controlled environment at 23 ± 1 °C with *ad libitum* access to water and a normal diet (MF, Oriental Yeast Co., Ltd.). Dose selection was guided by prior in vivo studies of Lac-Phe [13] and feasibility considerations for behavioral testing. Mice received a single dose of vehicle (saline) or Lac-Phe by oral gavage (100 or 300 µg/mouse; 4 or 12 mg/kg) or intraperitoneal injection (30 or 100 µg/mouse; 1.2 or 4 mg/kg) 2 h before the behavioral tests. These doses were used to define the dose range evaluated under our experimental conditions. For intracerebroventricular (ICV) injection studies, mice were infused with 1 μL of vehicle (saline) or Lac-Phe (1 µg or 10 μg/mouse) 2 h before the behavioral tests. All experimental procedures were conducted according to the ARRIVE guidelines.

### Cannula implantation and ICV injection

Mice were anesthetized with isoflurane and placed in a stereotaxic frame. A 26-gauge single stainless-steel guide cannula (C315GS-5-SPC, Plastics One, Roanoke, VA, USA) was implanted into the lateral ventricles (−0.45 mm from bregma, ±0.9 mm lateral and −2.5 mm from the skull). The cannula was fixed to the skull using screws and dental cement. Mice were housed in groups of six per cage and were allowed to recover from the operation in the subsequent week. The placement of the guide cannula was verified histologically at the end of the experiment. Mice were infused with 1 μL of saline or Lac-Phe (1 µg or 10 μg/mouse) 2 h before the behavioral tests. SCH23390 (1 μg/mouse) was administered 30 min before Lac-Phe injection.

### EPM test

The EPM test is widely used to measure anxiety-like behaviors in mice [19]. The maze (MK-10 system; Shin factory, Fukuoka, Japan) comprises two closed arms (30 cm length × 6 cm width × 15 cm height) and two open arms (30 cm length × 6 cm width) raised 50 cm above the floor. The head of the test mouse was placed at the center of the device, facing the open arm, and its behavior was observed for the next 6 min. The cumulative duration in the open arms, closed arms, and head-dip zone, the frequency of entry into each arm or zone, and the distance moved were measured using an EthoVision XT 15 system (Noldus Information Technology, Wageningen, The Netherlands/Sophia Scientific, Aichi, Japan). The EPM test started at 2:00 p.m. On experimental days, the mice were injected intraperitoneally or orally administered each solution (control (saline) or Lac-Phe (30, 100 or 300 µg/mouse) 2 h before the test.

### NSFT

NSFT is a behavioral test that considers an animal’s hesitation to consume highly palatable food in a novel environment as a measure of anxiety and loss of pleasure [20]. On experimental days, the mice were injected intraperitoneally with control (saline) or Lac-Phe (30 or 100 µg/mouse) 2 h before the test. The mice were fasted for 18 h prior to injection, with water available ad libitum. NSFT started at 1:30 p.m. In the open field, one tablet of normal chow was placed in the center area, and the test mouse was placed in the corner of the border area. The cumulative duration spent in the central area and distance traversed were measured using the EthoVision XT 15 system.

### Tail suspension test (TST)

TST was conducted following a previously reported protocol [21]. The 6 min test began immediately after the mouse was suspended and was recorded using a Stoelting USB camera mounted on a tripod and connected to a computer. The time that the animal spent immobile without trunk curling and with trunk curling was measured and added (total immobility duration) [22]. Total immobility duration was used as a measure of depression-like behavior.

### High-performance liquid chromatography (HPLC) analysis for dopamine levels

HPLC analysis was performed following a previously reported method with slight modifications [23, 24]. In brief, regional dopamine levels were measured in excised brain tissue by isocratic HPLC separation and electrochemical detection (ECD). Mice were euthanized 1 and 2 h after administration. The frozen brain tissue was homogenized in 0.2 mol/L perchloric acid and centrifuged at 20,000 × g at 0 °C for 15 min. The resulting supernatant was centrifuged at 17,800 × g for 5 min at 0 °C and filtered through a 0.45 μm filter. In this study, only dopamine from the supernatant was quantified using the HPLC-ECD system (Eicom Corporation, Kyoto, Japan). Dopamine levels in the brain tissue were determined using an HPLC system equipped with a C18 reverse-phase column (Eicompak SC-5ODS, 150 mm × 3.0 ID, Eicom Corporation, Kyoto, Japan), guard column (PC-04, Eicom Corporation, Kyoto, Japan), and column thermostat (ATC-300) set to 25 °C. Molecules were separated using a mixture of 0.1 mol/L acetic acid–citrate buffer (pH 3.5), methanol, 100 mg/mL sodium 1-octanesulfonate solution, and 5 mg/mL ethylenediaminetetraacetic acid-2Na solution (840:160:1.9:1) under the control of a pump system (EP-300) at a flow rate of 0.5 mL/min. For each measurement, 10 μL of the brain lysate supernatant was injected automatically using an automated sample injector. An electrochemical detector (ECD-300) with a graphite electrode (WE-3G), gasket, and Ag/AgCl reference electrode set to +750 mV was used for quantification.

### Measurement of serum corticosterone levels

Mice rested for 1 or 2 h after intraperitoneal administration of Lac-Phe and were subsequently subjected to restraint stress using a custom-built restrainer trap (16 × 11 cm) for 60 min. Mice were rested for 30 min after the restraint-stress treatment, followed by the collection of blood samples for measuring corticosterone levels. Blood samples were allowed to stand overnight at 4 °C and were centrifuged on the following day at 1000 × g for 15 min at 4 °C. The resulting supernatants were centrifuged again at 3440 × g for 5 min at 4 °C, and the final supernatants were used as serum samples and stored at −80 °C [25]. Serum corticosterone levels, as a stress marker, were determined using an enzyme immunoassay kit (TECAN, The IBL Corticosterone Enzyme Immunoassay Kit, RE52211, Germany) following the manufacturer’s guidelines.

### Total RNA extraction and quantitative real-time polymerase chain reaction (PCR)

Quantitative real-time PCR was performed using a previously reported method [26]. Hippocampal samples were collected from C57BL/6 mice administered saline or Lac-Phe 2 h before euthanasia. Total RNA was extracted using an RNeasy Mini Kit (Qiagen, Hilden, Germany). Complementary DNA was generated using the Takara Prime Script® RT Master Mix (Takara, Osaka, Japan). For quantitative real-time PCR, the cDNA sequence was identified on the CFX Connect Real-Time PCR Detection System (Bio-Rad Laboratories, Inc., California, USA) with the THUNDERBIRD® qPCR Mix (Toyobo Co., Osaka, Japan), with primers designed specifically for mouse IL1β, IL6, and TNFα. A total of 40 cycles of denaturation at 95 °C for 15 s and annealing and elongation at 60 °C for 60 s were performed. Normalized mRNA levels were expressed in arbitrary units obtained by dividing the average, efficiency-corrected values for sample mRNA expression by those for β-actin RNA expression for each sample. The resulting values were expressed as fold-change over the average control levels. The following primer sequences were used: IL-1β (F-GCAACTGTTCCTGAACTCAACT and R-ATCTTTTGGGGTCCGTCAACT), IL6 (F-TAGTCCTTCCTACCCCAATTTCC and R-TTGGTCCTTAGCCACTCCTTC), TNFα (F-CCCTCACACTCAGATCATCTTCT and R-GCTACGACGTGGGCTACAG), or β-actin (F-CTGCGCAAGTTAGGTTTTGTCA and R-TGCTTCTAGGCGGACTGTTACTG).

### TGFα shedding assay

The TGFα shedding assay was performed in HEK293 cells (ThermoFisher Scientific, R70507) as described previously with minor modifications [27]. Cells were routinely tested for mycoplasma contamination, and all tests were negative. Plasmid transfection was performed in a 6 cm dish with a mixture of 1000 ng (per well in a 6 cm dish) AP-TGFα-encoding plasmid, 400 ng GPCR-encoding plasmid and 40 ng each of eight wild-type or chimeric Gα subunits (Gα_q/s_, Gα_q/i1_, Gα_q/i3_, Gα_q/o_, Gα_q/z_, Gα_q/12_, Gα_q/13_ and Gα_16_). After 1-day culture, the transfected cells were harvested by trypsinization, pelleted by centrifugation at 190 × g for 5 min, and washed once with 5 mM HEPES (pH 7.4)-containing Hank’s Balanced Salt Solution (HBSS). After centrifugation, the cells were resuspended in 16 mL of the HEPES-containing HBSS. The cell suspension was seeded in a 96-well culture plate (cell plate) at a volume of 90 μL (per well hereafter) and incubated in a 37 °C incubator with 5% CO_2_ for 30 min. The cells were mixed with 10 µL of 10 × Lac-Phe (diluted in HBSS containing 5 mM HEPES (pH 7.4) and 0.01% (w/v) fatty acid-free BSA (Serva)), and incubated for 60 min. After spinning the cell plates at 190 × g for 2 min, 80 μL of conditioned media was transferred to an empty 96-well plate (conditioned media (CM) plate). Then, 80 μL of AP reaction solution (10 mM *p*-nitrophenylphosphate (*p*-NPP), 120 mM Tris-HCl (pH 9.5), 40 mM NaCl, and 10 mM MgCl_2_) was dispensed into the cell plates and the CM plates. Absorbance at 405 nm of the plates was measured using a microplate reader (SpectraMax 340 PC384, Molecular Devices) before incubation and after 1-h and 2-h of incubation at room temperature. Ligand-induced AP-TGFα release was calculated by subtracting spontaneous AP-TGFα release signal from ligand-induced AP-TGFα release signal.

### LC-MS analysis of Lac-Phe

The samples were acidified with 6 N HCl. Then, the samples were extracted with ethyl acetate (100 µL × 3) and the combined ethyl acetate layer was evaporated with N_2_ gas. The extracts were dissolved in H_2_O containing 1% AcOH (1 mL). These solutions were loaded onto a WAX cartridge column (1 cc, 30 mg, Waters), washed with H_2_O containing 1% AcOH (1 mL), 80% MeCN (2 mL) and 80% MeCN containing 1% AcOH (2 mL) and then eluted with 80% MeCN containing 1% HCOOH (2 mL). The 1% HCOOH eluted fractions were concentrated and dissolved in MeCN. The samples were subjected to LC–MS/MS to analyze Lac-Phe. LC–MS/MS analysis of Lac-Phe was conducted using a quadrupole/time-of-flight tandem mass spectrometer (X500R, AB SCIEX) and an ultrahigh performance liquid chromatography (Nexera, Shimadzu) equipped with a reverse-phase column (CORTECS UPLC phenyl, 1.6 µm, φ 2.1 × 75 mm; Waters). For Lac-Phe analysis, the elution of the samples was carried out with H_2_O containing 0.05% AcOH (solvent A) and MeCN containing 0.05% AcOH (solvent B), and the mobile phase was changed from 2% B to 98% at 4.5 min after the injection, respectively, at a flow rate of 0.3 mL/min. The MS analysis conditions were as follows: negative-ion mode; declustering potential, –80 V; collision energy, –15 V; and parent ions (m/z) of 236.09. Peak areas were calculated using a fragment ion, 88.04.

### Statistical analysis

All data are expressed as mean ± SEM. Statistical analyses were performed using a two-tailed unpaired Student’s t-test or one-way ANOVA followed by post hoc Tukey’s or Dunnett’s tests. All statistical analyses were performed using Prism version 10 (GraphPad Software, San Diego, CA, USA). Differences were considered statistically significant at p < 0.05. Sample sizes were chosen based on our previous experience with these assays and similar published studies; no a priori sample size calculation was performed. Animals were allocated to groups after matching for age and body weight; a formal randomization sequence was not used. Investigators were not blinded to group allocation during the experiments. A priori exclusion criteria included incorrect cannula placement, postoperative complications, or technical failure preventing outcome assessment. For animal experiments, the experimental unit was an individual mouse (biological replicate), and the final n for each group is provided in the corresponding figure legends. Assumptions for parametric analyses (normality and homogeneity of variance) were assessed in Prism 10, and the datasets analyzed met the assumptions for the tests applied.

### Study approval

The Animal Research Committee of Meiji University (MUIACUC2022-05) (Kanagawa, Japan) reviewed and approved all animal experiments. All experiments were conducted in accordance with the regulations of Meiji University and ARRIVE guidelines.

## Results

### Oral administration of Lac-Phe elicited anxiolytic-like effects

**Fig. 1:**
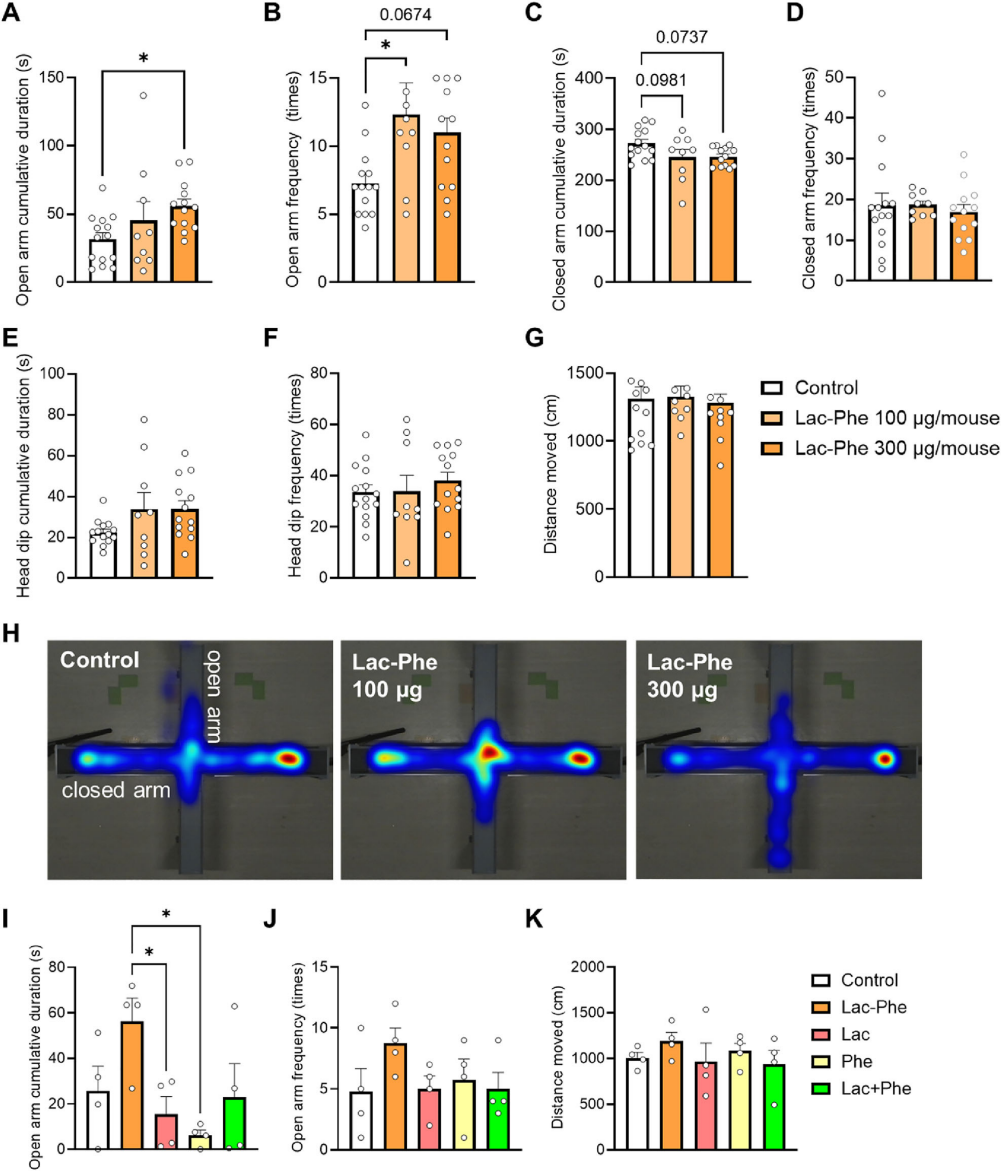
Oral administration of Lac-Phe exerts anxiolytic-like effects. **A–H** Mice were orally administered vehicle (saline, n = 14), Lac-Phe 4 mg/kg (approximately 100 μg/mouse, n = 9), or Lac-Phe 12 mg/kg (approximately 300 μg/mouse, n = 13). Open arm cumulative duration **A**, open arm frequency **B**, closed arm cumulative duration **C**, closed arm frequency **D**, head dip cumulative duration **E**, head dip frequency **F**, total distance moved **G**, and representative heat map **H** during the 6-min elevated plus maze test. **I–K** The mice were orally administered vehicle (saline, n = 4), Lac-Phe (n = 4), Lac (n = 4), Phe (n = 4), or Lac+Phe (n = 4). Each group was administered 50 μmol/kg. Open arm cumulative duration **I**, open arm frequency **J**, and total distance moved **K**, during the 6-min elevated plus maze test. *p < 0.05 for one-way ANOVA followed by Dunnett’s multiple comparisons tests in **A** and **B**, and Tukey’s multiple comparison tests in **I**. Data represent the mean ± SEM.

Lac-Phe was orally administered to mice to investigate its anxiolytic-like effects. Oral Lac-Phe treatment significantly increased the open arm cumulative duration (Fig. 1A), showing no significant difference in the distance moved (Fig. 1G) during the EPM test. The open arm frequency increased significantly (Fig. 1B), while the closed arm cumulative duration showed a tendency to decrease in Lac-Phe-treated mice (Fig. 1C). Closed arm frequency showed no significant difference (Fig. 1D). The head dip cumulative duration and frequency were defined as the number of times the mouse looked down in the open arm area and were slightly increased in the Lac-Phe group (300 μg/mouse) compared with those in the control group (Fig. 1E, F). Heatmap analysis suggested that Lac-Phe-treated mice remained longer in the open arms (Fig. 1H). In addition, LC–MS analysis showed an increased Lac-Phe-derived signal in portal vein plasma 15 min after oral administration, indicating that orally administered Lac-Phe can reach the portal circulation under our experimental conditions (Fig. S1). Collectively, these findings indicate a dose-related increase in anxiolytic-like behavior after oral Lac-Phe administration, with partial activity at 100 μg/mouse and a more robust effect at 300 μg/mouse.

We tested the structural importance of Lac-Phe in anxiolytic-like behavior following oral administration. The only significant effect of oral Lac-Phe treatment was an increase in open arm cumulative duration (Fig. 1I), with no significant differences in total distance moved (Fig. 1K) or the open arm frequency (Fig. 1J) during the EPM test. Thus, oral Lac-Phe selectively exerts anxiolytic-like effects in mice without affecting the total distance moved. Our findings demonstrate that only Lac-Phe, and not Lac or Phe alone, exerts anxiolytic-like effects in mice following oral administration.

### Intraperitoneal administration of Lac-Phe exerted anxiolytic-like effects in the EPM test and NSFT

**Fig. 2:**
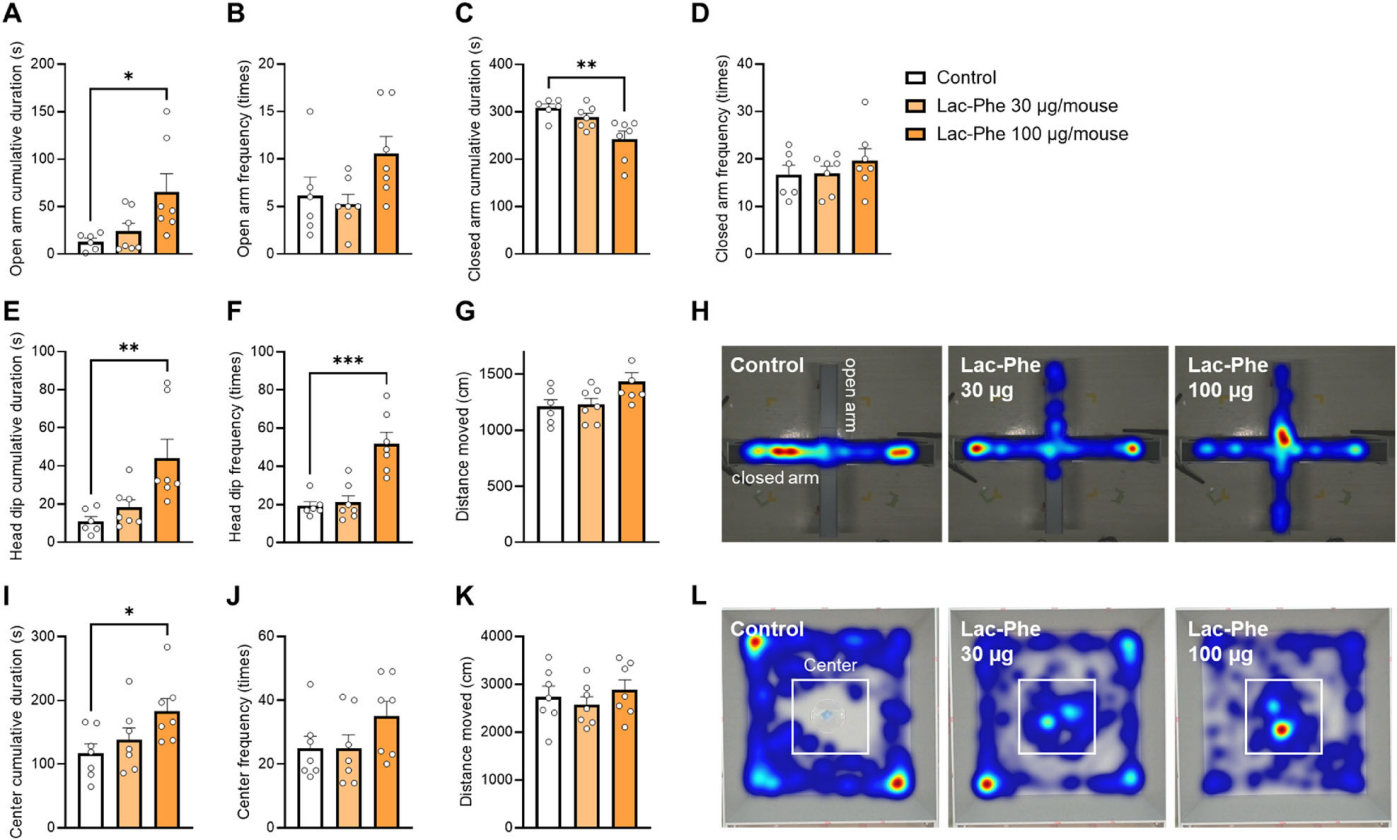
Intraperitoneal administration of Lac-Phe exerts anxiolytic-like effects in the elevated plus maze test and novelty-suppressed feeding test. **A–H** Mice were intraperitoneally administered vehicle (saline, n = 6), Lac-Phe 1.2 mg/kg (approximately 30 μg/mouse, n = 7), or Lac-Phe 4 mg/kg (approximately 100 μg/mouse, n = 7) for the EPM. Open arm cumulative duration **A**, open arm frequency **B**, closed arm cumulative duration **C**, closed arm frequency **D**, head dip cumulative duration **E**, head dip frequency **F**, total distance moved **G**, and representative heat map **H** during the 6-min elevated plus maze test. **I–L** Mice received intraperitoneal injections of vehicle (saline, n = 7), Lac-Phe 1.2 mg/kg (approximately 30 μg/mouse, n = 7), or 4 mg/kg (approximately 100 μg/mouse, n = 7) for the novelty-suppressed feeding test. Center cumulative duration **I**, center frequency **J**, total distance moved **K**, and representative heat map **L** during the 10-min novelty-suppressed feeding test. *p < 0.05, **p < 0.01, ***p < 0.001 for one-way ANOVA, followed by Dunnett’s multiple comparison tests in **A,** **C,** **E,** **F**, and **I**. Data represent the mean ± SEM.

To determine whether intraperitoneal administration of Lac-Phe exhibited anxiolytic-like effects similar to those observed after oral administration, we intraperitoneally administered Lac-Phe (30 or 100 µg/mouse) or vehicle (saline) and performed the EPM test. As shown in Fig. 2, acute intraperitoneal administration of Lac-Phe induced a significant increase in the cumulative duration spent in the open arms (Fig. 2A). There was no significant difference in distance moved (Fig. 2G) or the open arm frequency (Fig. 2B). Head dip cumulative duration spent and head dip frequency in the open arms increased significantly (Fig. 2E, F) in the Lac-Phe-treated groups. Conversely, Lac-Phe treatment decreased cumulative duration in the closed arms significantly compared with that in controls (Fig. 2C), with no significant difference in the closed arm frequency among the groups (Fig. 2D). Heatmap analysis showed that the Lac-Phe-treated mice remained longer in the open arms (Fig. 2H). Thus, intraperitoneal administration of Lac-Phe increased the time of stay in the open arm without affecting the distance moved, suggesting that intraperitoneal administration of Lac-Phe also exerted anxiolytic-like effects in mice.

To assess the anxiolytic-like potential of Lac-Phe, we conducted an additional anxiety-related behavioral test. Along with the EPM, NSFT, a paradigm commonly used to assess the activity of anxiolytic- or antidepressant-like compounds in mice, was performed. On the experimental day, NSFT was conducted 2 h after the intraperitoneal administration of Lac-Phe (30–100 μg). The cumulative duration spent in the center significantly increased in the Lac-Phe-treated groups (Fig. 2I), whereas the total distance moved showed no significant change (Fig. 2K). The frequency of center entries was slightly higher in the Lac-Phe-treated groups than in the control (Fig. 2J). Heat map analysis further confirmed that Lac-Phe-treated mice remained longer in the center (Fig. 2L). These results demonstrate that intraperitoneal Lac-Phe administration exerts anxiolytic-like effects across multiple behavioral paradigms in mice.

### Central administration of Lac-Phe exhibited anxiolytic-like effects

**Fig. 3:**
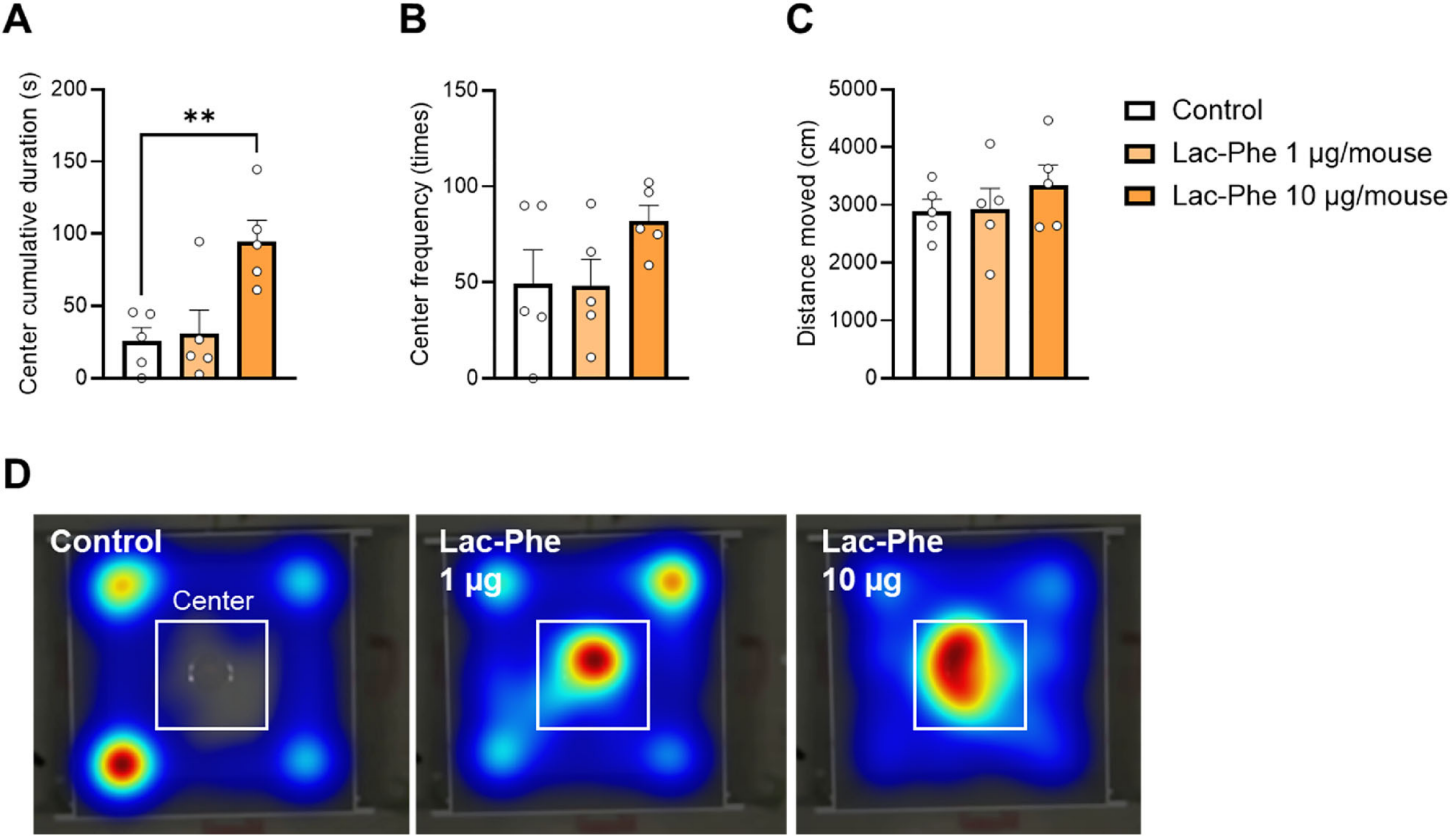
Central administration of Lac-Phe exerts anxiolytic-like effects in mice. **A–D** Mice were centrally administered vehicle (saline, n = 6), Lac-Phe 1 μg/mouse (n = 7), or Lac-Phe 10 μg/mouse (n = 6). Center cumulative duration **A**, center frequency **B**, total distance moved **C**, and representative heat map **D** during the 10-min novelty-suppressed feeding test. **p < 0.01 for one-way ANOVA followed by Dunnett’s multiple comparison tests in **A**. Data represent the mean ± SEM.

Numerous studies have demonstrated the crucial role of the central nervous system in regulating anxiety behavior; therefore, we investigated whether Lac-Phe produces anxiolytic-like effects after ICV administration. As shown in Fig. 3, ICV injection of Lac-Phe significantly increased the center cumulative duration in the Lac-Phe-treated group (Fig. 3A), with no significant difference in the distance moved (Fig. 3C). The frequency of entering the center was slightly increased in the Lac-Phe-treated group compared to that in the control (Fig. 3B). Heatmap analysis showed that the Lac-Phe-treated mice remained longer in the center (Fig. 3D). ICV administration of Lac-Phe also produced greater anxiolytic-like effects than either lactate or phenylalanine (Fig. S2). Our results demonstrate that Lac-Phe exerts more potent anxiolytic-like effects than lactate or phenylalanine when administered centrally.

### Blockade of central dopamine D1 and serotonin 5-HT1A receptor systems abolished Lac-Phe-induced anxiety-like behavior

**Fig. 4:**
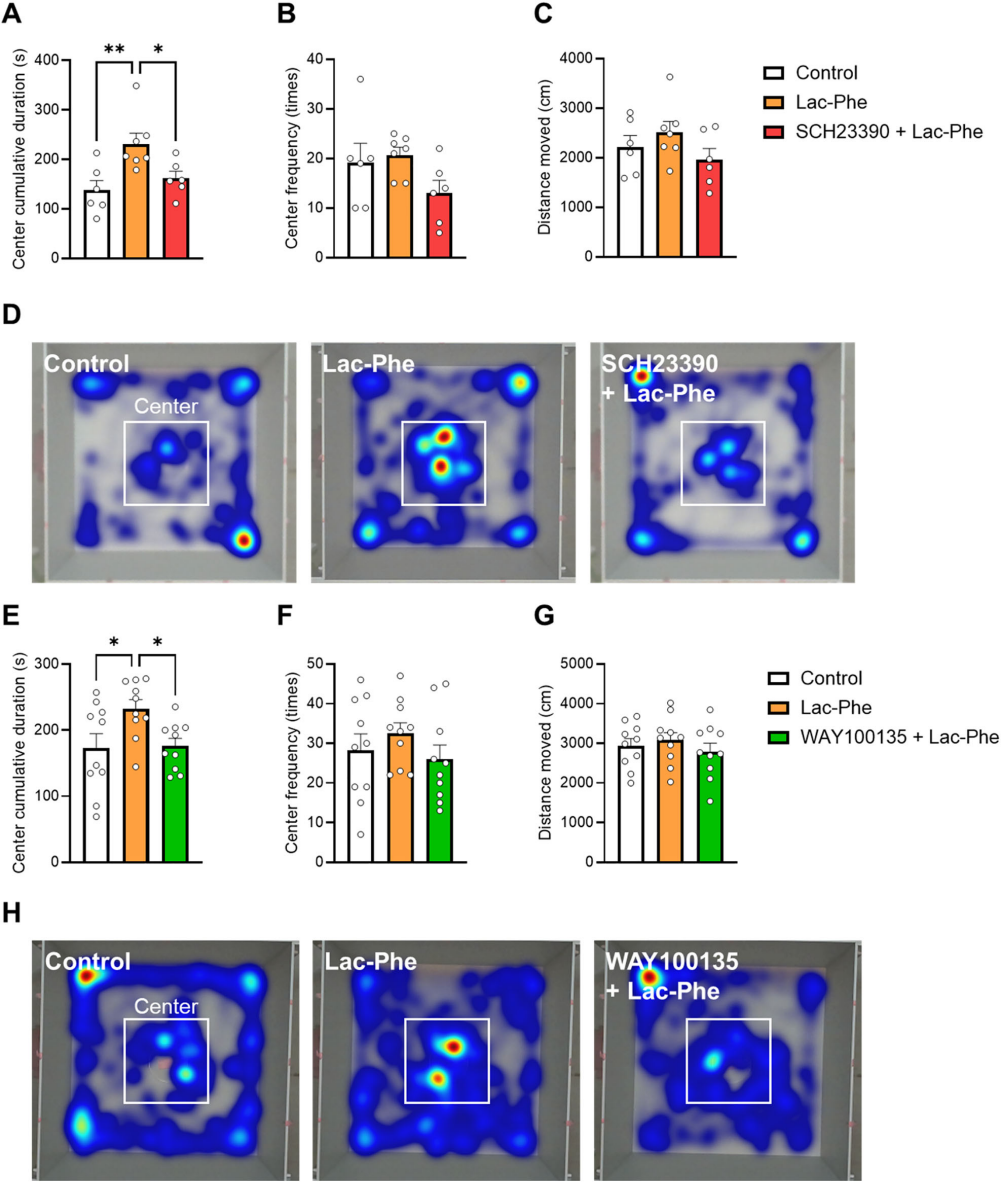
Blockade of central dopamine D1 and serotonin 5-HT1A receptor systems abolishes Lac-Phe-induced anxiety-like effects. **A–D** Mice were centrally administered vehicle (saline) or SCH23390 (1 μg/brain/mouse). Subsequently, 30 min after antagonist injection, the mice were intraperitoneally administered vehicle or Lac-Phe 4 mg/kg (100 μg/mouse). Group sizes were as follows: vehicle + vehicle, n = 6; vehicle + Lac-Phe, n = 7; SCH23390 + Lac-Phe, n = 6. Center cumulative duration **A**, center frequency **B**, total distance moved **C**, and representative heat map **D** during the 10-min novelty-suppressed feeding test. **E–H** The mice were intraperitoneally administered vehicle (saline) or WAY100135 (250 μg/mouse). Subsequently, 30 min after antagonist injection, the mice were intraperitoneally administered vehicle or Lac-Phe 4 mg/kg (100 μg/mouse). Group sizes were as follows: vehicle + vehicle, n = 10; vehicle + Lac-Phe, n = 10; WAY100135 + Lac-Phe, n = 10. Center cumulative duration **E**, center frequency **F**, total distance moved **G**, and representative heat map **H** during the 10-min novelty-suppressed feeding test. *p < 0.05, **p < 0.01 for one-way-ANOVA followed by Tukey’s multiple comparison tests in **A** and **E**. Data represent the mean ± SEM.

Dopamine and serotonin are key neurotransmitters regulating anxiety. We examined the effects of receptor antagonists to investigate whether these systems contribute to the anxiolytic-like effects of Lac-Phe. Central administration of SCH23390, a D1 receptor antagonist, significantly blocked the Lac-Phe–induced increase in center duration in the NSFT, without affecting the total distance moved (Fig. 4A–C). Similarly, intraperitoneal administration of WAY100135, a selective 5-HT1A receptor antagonist, abolished the Lac-Phe–induced increases in center duration, with no changes in distance moved (Fig. 4E–G). Heatmap analysis supported these findings by showing prolonged center occupancy in Lac-Phe-treated mice (Fig. 4D, H). These results suggest that the anxiolytic-like effects of Lac-Phe are consistent with the involvement of central dopaminergic and serotonergic signaling.

### Lac-Phe enhanced dopamine levels in the striatum and hypothalamus

**Fig. 5:**
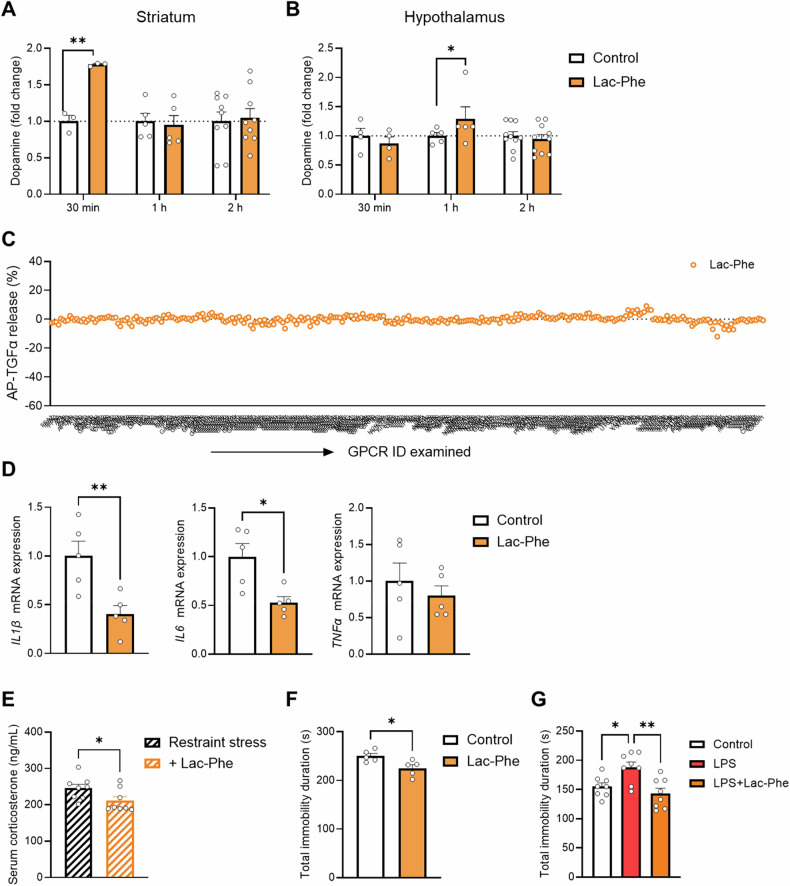
Lac-Phe enhances tissue dopamine levels in the striatum and hypothalamus. **A, B** Dopamine levels in the striatum **A** and hypothalamus **B** dissected at 30 min, 1 h, and 2 h following intraperitoneal administration of vehicle (saline) or Lac-Phe (100 μg/mouse). Group sizes were as follows: **A** striatum: 30 min, vehicle n = 3 and Lac-Phe n = 3; 1 h, vehicle n = 5 and Lac-Phe n = 5; 2 h, vehicle n = 9 and Lac-Phe n = 9. **B** hypothalamus: 30 min, vehicle n = 4 and Lac-Phe n = 4; 1 h, vehicle n = 5 and Lac-Phe n = 5; 2 h, vehicle n = 10 and Lac-Phe n = 10. **C** Pharmacological screening of Lac-Phe agonist activity among 310 GPCRs using the TGFα shedding assay, performed in technical triplicate. **D** Hippocampal mRNA expression of *IL-1β*, *IL-6*, and *TNFα* was measured 2 h after intraperitoneal administration of vehicle (saline, n = 5) or Lac-Phe (100 μg/mouse, n = 5). **E** Serum corticosterone levels in restraint-stressed mice administered with vehicle (saline, n = 8) or Lac-Phe 4 mg/kg (approximately 100 μg/mouse, n = 8). **F** Total immobility duration during the 6-min tail suspension test. Mice were intraperitoneally administered vehicle (saline, n = 5) or Lac-Phe 4 mg/kg (approximately 100 μg/mouse, n = 5) for 3 consecutive days. **G** Total immobility duration during the 6-min tail suspension test. Mice were intraperitoneally administered vehicle (saline) or LPS (0.8 mg/kg) followed by saline or Lac-Phe 4 mg/kg (approximately 100 μg/mouse) for 2 days. Group sizes were as follows: vehicle + vehicle, n = 8; LPS + vehicle, n = 8; LPS + Lac-Phe, n = 8. *p < 0.05, **p < 0.01 for t-tests in **A,** **B,** **D,** **E**, and **F**, and one-way-ANOVA followed by Tukey’s multiple comparison tests in **G**. Data represent the mean ± SEM.

The striatum and hypothalamus are critical brain regions that regulate emotional behaviors through monoamine signaling [28]. Lac-Phe intraperitoneal administration increased tissue dopamine levels in these regions, with a rapid increase in the striatum at 30 min and a delayed increase in the hypothalamus at 1 h (Fig. 5A, B). Although these measurements do not directly capture synaptic dopamine release or neurotransmission, these temporally distinct changes were observed following Lac-Phe administration and may reflect altered dopaminergic-related measures in these regions. Given that impaired dopaminergic signaling has been implicated in anxiety- and mood-related phenotypes, these data are consistent with a potential contribution of dopaminergic mechanisms to the anxiolytic-like effects of Lac-Phe.

To investigate potential GPCR targets of Lac-Phe, we performed a TGFα shedding assay screening covering 310 GPCRs, including dopamine receptors. Using a concentration of 100 µM Lac-Phe, we observed no significant agonist activity across the panel of GPCRs. Importantly, these negative findings do not exclude receptor-mediated actions, because Lac-Phe may act indirectly, require accessory factors not present in the assay system, or signal via non-GPCR targets, transporters, or downstream metabolites (Fig. 5C). Accordingly, at least two non-mutually exclusive explanations may account for the lack of detectable activity in this assay: First, Lac-Phe may exert its effects through indirect mechanisms via receptors not represented in this screening (Fig. 5C). Second, while the TGFα shedding assay is capable of detecting activation for >90% of known liganded GPCRs, Lac-Phe could potentially activate GPCRs that are incompatible with this assay system, including certain orphan GPCRs whose signaling properties remain poorly characterized.

In addition to modulating monoamine production, Lac-Phe suppressed neuroinflammatory responses in the brain. Hippocampal expression of IL-1β and IL-6 was significantly reduced, and TNFα expression showed a downward trend following Lac-Phe treatment (Fig. 5D). Because neuroinflammation is increasingly being recognized as a driver of stress vulnerability, cognitive decline, and psychiatric symptomatology, the ability of Lac-Phe to dampen pro-inflammatory cytokine expression suggests a protective role against stress-induced neuropathology. Thus, Lac-Phe is associated with reduced expression of inflammatory markers in the brain.

Consistent with these findings, Lac-Phe pretreatment reduced the corticosterone surge induced by acute restraint stress (Fig. 5E). Corticosterone is a well-established stress hormone, and elevated levels are closely associated with anxiety- and depression-related states [29, 30]. Because chronic stress often leads to depression-like behavior [31], we next examined whether Lac-Phe influenced depression-related phenotypes. In the tail suspension test (TST), Lac-Phe significantly reduced immobility, reflecting increased motivation-related behavior (Fig. 5F). Lac-Phe effectively counteracted the prolonged immobility induced by LPS, a model of inflammation-related depression [32] (Fig. 5G). These results indicate that Lac-Phe can alleviate depressive-like states under both physiological and pathological conditions, reinforcing its broader role as a modulator of mood-related behavior.

These findings highlight Lac-Phe as a bioactive metabolite that modulates emotional behavior and is associated with changes in monoaminergic signaling, inflammatory markers, and stress hormone response. Although additional work is required to identify the proximal mediator(s) and to define pharmacokinetics and tissue exposure, our results uncover a previously underappreciated link between Lac-Phe and anxiety-related behavior in mice.

## Discussion

In this study, we found that Lac-Phe administration was associated with anxiolytic-like behavioral effects in mice, as assessed by the EPM and NSFT. The behavioral effects were observed under oral dosing conditions and were also reproducible after intraperitoneal and intracerebroventricular administration, supporting the robustness of the phenotype across administration routes. Pharmacological blockade experiments further suggested the involvement of dopamine D1 and serotonin 5-HT1A receptor signaling in these effects.

Lac-Phe is an endogenous metabolite whose plasma concentration increases following physical exercise. Lac-Phe administration in obese animal models suppresses food intake and induces weight loss, suggesting potential metabolic benefits and raising the possibility of CNS-related involvement. However, the broader implications of Lac-Phe for brain-related outcomes remain to be defined, including how different dosing routes and tissue exposure relate to these effects. Our findings extend the functional scope of Lac-Phe to anxiety-related behavior and motivate future studies to identify the proximal mediator(s) and to characterize pharmacokinetics and tissue exposure, particularly under oral dosing conditions. To our knowledge, this is the first study to link Lac-Phe to anxiety-related behavior in mice, with effects observed under oral dosing conditions and recapitulated by intraperitoneal and intracerebroventricular administration.

Anxiety, characterized by worry and tension, is a common emotional state, and chronic stress increases the risk of psychiatric disorders. EPM and NSFT are widely used to evaluate anxiolytic efficacy in rodents. Our findings revealed that Lac-Phe consistently increased open-arm exploration in the EPM and promoted center exploration in the NSFT, without altering locomotor activity. This behavioral profile indicates genuine anxiolytic-like activity rather than nonspecific hyperactivity. Moreover, since the anorexigenic effect of Lac-Phe is observed only in obese animals, the anxiolytic-like effects observed in the NSFT are unlikely to reflect appetite suppression.

Monoaminergic neurotransmitters, such as dopamine and serotonin, play crucial roles in emotional regulation. In this study, pharmacological blockade of dopamine D1 or serotonin 5-HT1A receptors attenuated the anxiolytic-like effects of Lac-Phe, suggesting that these pathways contribute to the observed behaviors. Consistently, Lac-Phe administration increased tissue dopamine levels in the striatum and hypothalamus in a time-dependent manner, although tissue content does not directly indicate increased neurotransmitter release or synaptic transmission. In contrast, we did not detect significant changes in tissue serotonin or noradrenaline levels under the present conditions (Fig. S3); therefore, future work using complementary neurochemical and electrophysiological approaches will be important to clarify how serotonergic signaling may be engaged. In addition to monoamines, inhibitory and excitatory neurotransmission mediated by GABA and glutamate is well known to shape anxiety-related behaviors. Because we did not quantify GABA or glutamate in the present study, the potential contribution of altered inhibitory/excitatory balance to the Lac-Phe–associated behavioral effects remains unclear. Future studies should assess these neurotransmitter systems alongside monoamines to better define the circuit and neurochemical mechanisms involved. Notably, tissue dopamine content exhibited transient changes (striatal peak at 30 min and hypothalamic peak at 1 h), whereas behavioral testing was performed 2 h after administration. Because tissue dopamine measurements provide a snapshot of total content at the time of collection and do not directly reflect synaptic release or signaling dynamics, the lack of a difference at 2 h does not exclude earlier dopaminergic engagement that could contribute to the behavioral phenotype observed at 2 h. Behavioral outcomes may reflect integrated circuit-level effects and downstream signaling processes that can persist beyond transient changes in tissue monoamine content.

To further explore potential molecular targets of Lac-Phe, we conducted a broad GPCR screen using a TGFα shedding assay covering 310 receptors. Under the conditions tested (100 µM Lac-Phe), we did not detect significant agonist activity across the panel. Importantly, this negative result does not exclude receptor-mediated actions, because Lac-Phe may act indirectly, require accessory factors not present in the assay system, or signal via targets not captured by this format (e.g., non-GPCR targets, transporters, or downstream metabolites). Accordingly, the anxiolytic-like effects of Lac-Phe may involve indirect or alternative signaling mechanisms, and complementary approaches will be needed to clarify the proximal mediator(s) and molecular targets [33–35]. In addition, chemically stabilized Lac-Phe analogues have been described in patent literature to improve resistance to gastrointestinal degradation, underscoring the potential importance of pharmacokinetics and tissue exposure for oral delivery [35].

Beyond monoamine signaling, Lac-Phe attenuated neuroinflammation in the hippocampus by reducing the expression of IL-1β and IL-6, with a trend toward lower TNFα levels. Since elevated pro-inflammatory cytokines impair neurogenesis and synaptic plasticity and promote anxiety- and depression-like symptoms [36], the anti-inflammatory action of Lac-Phe may contribute to its stress-attenuating effects. Supporting this notion, Lac-Phe pretreatment significantly reduced corticosterone elevation induced by acute restraint stress, suggesting that it mitigates physiological stress responses. Given these stress-attenuating effects, we examined whether Lac-Phe could influence depression-related behavior. Beyond its anxiolytic-like properties, Lac-Phe reduced immobility in the TST, suggesting potential antidepressant-like effects. These results were observed both under basal conditions and in an inflammation-induced model, indicating that Lac-Phe may contribute to resilience against certain depression-like states. However, given the limited behavioral paradigms tested, further studies should establish whether these effects generalize to broader models of depression and clarify the underlying mechanisms. Because our experiments used an acute administration paradigm, the observed behavioral effects are unlikely to be explained solely by anti-inflammatory actions; instead, the cytokine changes may be most directly relevant to the LPS-induced condition, whereas basal-condition effects likely involve additional mechanisms, including monoamine-related signaling and stress hormone–related responses.

Lac-Phe is endogenously generated by the enzyme CNDP2, and its levels rise in the bloodstream after exercise [37]. While previous research has emphasized its metabolic actions, our findings uncover its neurological functions [38, 39], suggesting that Lac-Phe may contribute to the mental health benefits of physical activity, such as stress reduction and improved quality of life. It will be important to investigate the extent to which exercise-induced Lac-Phe mediates these psychological benefits and identify the specific underlying neural circuits.

We analyzed portal vein plasma 15 min after oral administration of Lac-Phe using an LC–MS-based method. The present portal vein data show that orally administered Lac-Phe can be detected in the portal circulation shortly after dosing. This finding supports the view that at least a fraction of orally administered Lac-Phe survives the gastrointestinal environment and is absorbed. However, these data do not by themselves establish systemic exposure, brain delivery, or the identity of the proximal mediator(s) underlying the behavioral effects. Behavioral testing was conducted 2 h after dosing as a standardized interval across administration routes and does not imply comparable exposure profiles between routes. Establishing validated pharmacokinetic and tissue exposure measurements after oral dosing remains an important priority for future work.

Lac-Phe is naturally present in certain fermented foods, such as Parmesan cheese and soy sauce, and can be produced by lactic acid bacteria found in kimchi [15]. Moreover, Lac-Phe may be obtained from underutilized protein-rich byproducts, such as whey. These characteristics position Lac-Phe as a candidate for development as a functional food ingredient with potential health-related and sustainability advantages. The possibility that dietary Lac-Phe derived from fermented foods is associated with stress- or anxiety-related outcomes warrants further investigation. Importantly, future studies will need to clarify the stability, bioavailability, and tissue exposure of Lac-Phe under dietary intake conditions. If these considerations can be addressed, Lac-Phe-enriched foods or beverages may represent a practical avenue to support stress- and anxiety-related well-being as part of the daily diet.

Other N-lactoyl amino acids (Lac-AAs) are naturally occurring compounds in various fermented foods, including Parmesan cheese [40], soy sauce [41, 42], and dry-cured ham [43], where they are generated through the action of lactoyl transferases in lactic acid bacteria. These Lac-AAs contribute to flavor attributes, such as mouthfeel [44] and kokumi taste [42], and have also been explored for applications in nutrition and health [45, 46]. The presence of Lac-Phe alongside other Lac-AAs in traditional foods highlights its potential as a naturally occurring food-derived bioactive compound. Therefore, Lac-Phe may not only act as a mediator of the mental health benefits of exercise but also represents a promising candidate for the development of functional foods or supplements that support emotional well-being.

In summary, our study shows that Lac-Phe administration elicits anxiolytic-like behavior in mice and that dopamine D1 receptor signaling and serotonin 5-HT1A receptor signaling contribute to these effects. Lac-Phe was also associated with changes in tissue dopamine levels, hippocampal inflammatory cytokine expression, and stress hormone responses. Collectively, our findings indicate that Lac-Phe merits further investigation as a food-derived metabolite with potential relevance to stress- and anxiety-related well-being, offering new perspectives for nutritional science and functional food development.

## Supplementary information

Supplemental Figure 1

Supplemental Figure 2

Supplemental Figure 3

Data Set 1

## Data Availability

Data are provided within the manuscript or supplementary information files. The datasets used and/or analyzed in this study are available from the corresponding author upon reasonable request.
