# Supplementary material for: Lac-Phe elicits anxiolytic-like effects associated with monoaminergic signaling in mice
Source: Transl Psychiatry. 2026 May 29;16:383. doi: 10.1038/s41398-026-04106-2 (PMC13408088; doi:10.1038/s41398-026-04106-2)
Supplement: Supplementary file 1 — Supplemental Figure 1 [file 41398_2026_4106_MOESM1_ESM.pptx]

## Slide 1
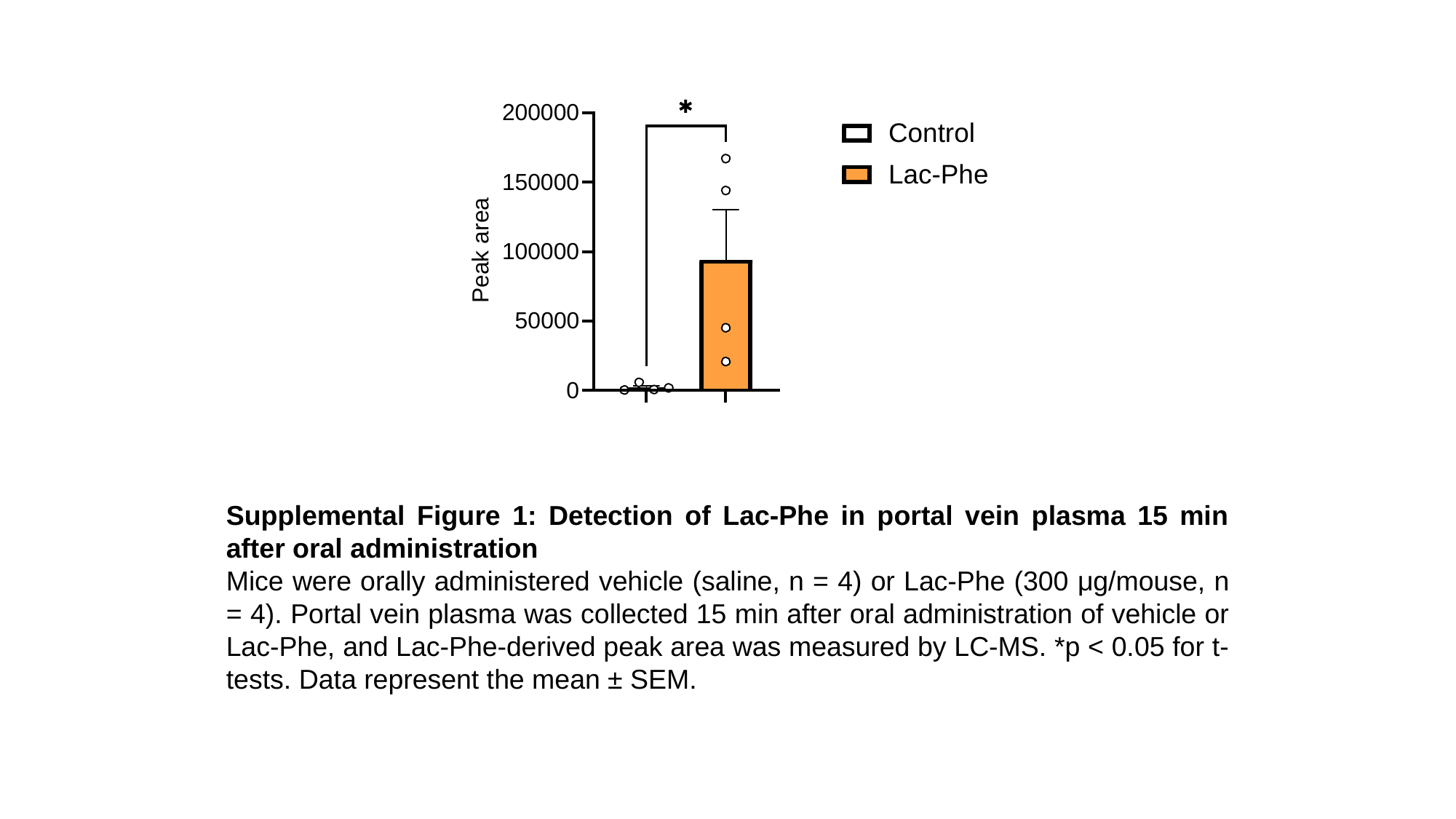

Supplemental Figure 1: Detection of Lac-Phe in portal vein plasma 15 min after oral administration
Mice were orally administered vehicle (saline, n = 4) or Lac-Phe (300 μg/mouse, n = 4). Portal vein plasma was collected 15 min after oral administration of vehicle or Lac-Phe, and Lac-Phe-derived peak area was measured by LC-MS. *p < 0.05 for t-tests. Data represent the mean ± SEM.
