# Supplementary material for: Lac-Phe elicits anxiolytic-like effects associated with monoaminergic signaling in mice
Source: Transl Psychiatry. 2026 May 29;16:383. doi: 10.1038/s41398-026-04106-2 (PMC13408088; doi:10.1038/s41398-026-04106-2)
Supplement: Supplementary file 2 — Supplemental Figure 2 [file 41398_2026_4106_MOESM2_ESM.pptx]

## Slide 1
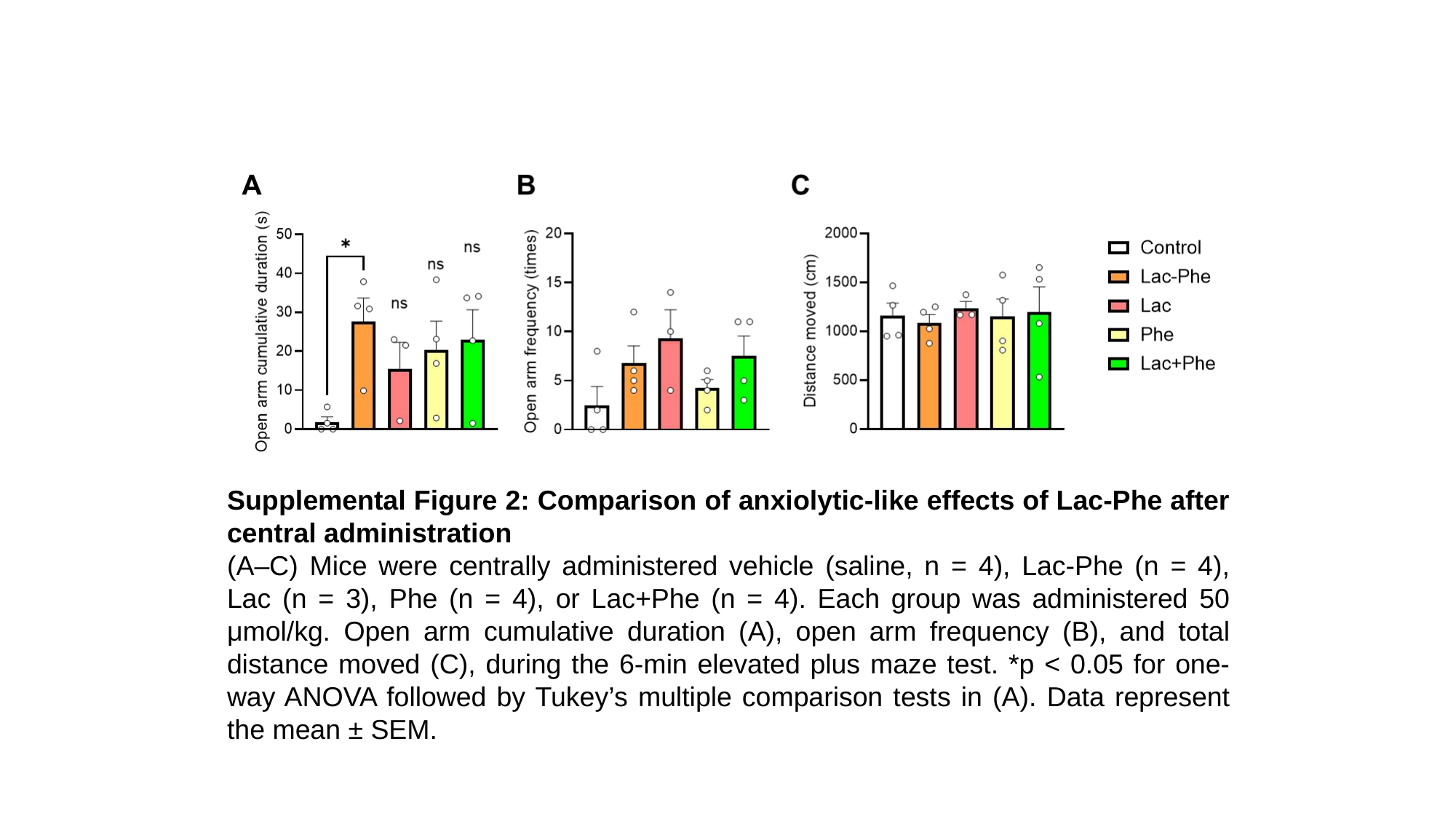

Supplemental Figure 2: Comparison of anxiolytic-like effects of Lac-Phe after central administration
(A–C) Mice were centrally administered vehicle (saline, n = 4), Lac-Phe (n = 4), Lac (n = 3), Phe (n = 4), or Lac+Phe (n = 4). Each group was administered 50 μmol/kg. Open arm cumulative duration (A), open arm frequency (B), and total distance moved (C), during the 6-min elevated plus maze test. *p < 0.05 for one-way ANOVA followed by Tukey’s multiple comparison tests in (A). Data represent the mean ± SEM.
