# Supplementary material for: Lac-Phe elicits anxiolytic-like effects associated with monoaminergic signaling in mice
Source: Transl Psychiatry. 2026 May 29;16:383. doi: 10.1038/s41398-026-04106-2 (PMC13408088; doi:10.1038/s41398-026-04106-2)
Supplement: Supplementary file 3 — Supplemental Figure 3 [file 41398_2026_4106_MOESM3_ESM.pptx]

## Slide 1
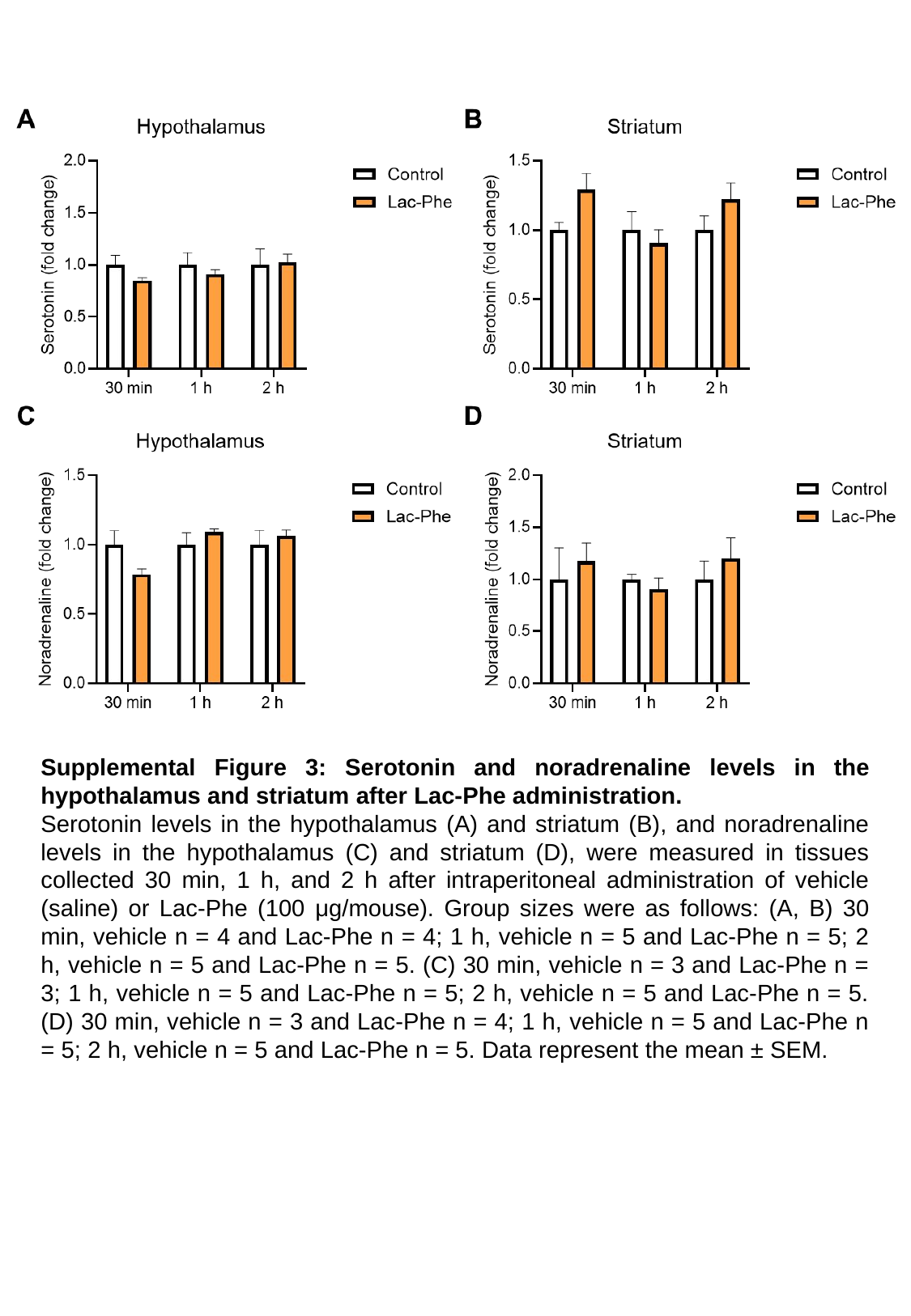

Supplemental Figure 3: Serotonin and noradrenaline levels in the hypothalamus and striatum after Lac-Phe administration.
Serotonin levels in the hypothalamus (A) and striatum (B), and noradrenaline levels in the hypothalamus (C) and striatum (D), were measured in tissues collected 30 min, 1 h, and 2 h after intraperitoneal administration of vehicle (saline) or Lac-Phe (100 μg/mouse). Group sizes were as follows: (A, B) 30 min, vehicle n = 4 and Lac-Phe n = 4; 1 h, vehicle n = 5 and Lac-Phe n = 5; 2 h, vehicle n = 5 and Lac-Phe n = 5. (C) 30 min, vehicle n = 3 and Lac-Phe n = 3; 1 h, vehicle n = 5 and Lac-Phe n = 5; 2 h, vehicle n = 5 and Lac-Phe n = 5. (D) 30 min, vehicle n = 3 and Lac-Phe n = 4; 1 h, vehicle n = 5 and Lac-Phe n = 5; 2 h, vehicle n = 5 and Lac-Phe n = 5. Data represent the mean ± SEM.
