# Supplementary material for: Lac-Phe elicits anxiolytic-like effects associated with monoaminergic signaling in mice
Source: Transl Psychiatry. 2026 May 29;16:383. doi: 10.1038/s41398-026-04106-2 (PMC13408088; doi:10.1038/s41398-026-04106-2)
Supplement: Supplementary file 4 — Data Set 1 [file 41398_2026_4106_MOESM4_ESM.pdf]

GPCR screening for Lac-Phe using the TGFα shedding assay.

No		AP-TGFα release (%)		
1	**5-HT1A**	6.4	±	0.47
2	**5-HT1B**	-0.22	±	0.51
3	**5-HT1D**	0.59	±	0.75
4	**5-HT1E**	-0.72	±	0.52
5	**5-HT1F**	0.31	±	0.17
6	**5-HT2A**	-0.68	±	0.39
7	**5-HT2B**	-0.48	±	0.43
8	**5-HT2C**	2.36	±	1.98
9	**5-HT3A**	-1.53	±	0.26
10	**5-HT3B**	0.09	±	0.52
11	**5-HT3C**	1.64	±	0.18
12	**5-HT4**	-0.4	±	0.14
13	**5-HT5A**	-0.47	±	0.51
14	**5-HT6**	0.34	±	0.72
15	**5-HT7**	1.85	±	0.17
16	**A1**	0.45	±	0.54
17	**A2A**	-0.53	±	0.23
18	**A2B**	-1.34	±	0.42
19	**A3**	-0.83	±	0.2
20	**APLNR**	0.63	±	0.83
21	**AT1**	-1.67	±	0.74
22	**AT2**	0.27	±	0.25
23	**B1**	-1.06	±	0.79
24	**B2**	-1.56	±	0.48
25	**BLT1**	0.1	±	0.24
26	**BLT2**	-1.92	±	0.51
27	**C3aR1**	-0.46	±	0.18
28	**C5aR1**	1.1	±	0.16
29	**CALCR**	0.29	±	0.23
30	**CALCRL**	-2.08	±	0.34
31	**CASR**	-0.13	±	0.12
32	**CB1**	0.9	±	0.24
33	**CB2**	1.01	±	0.46
34	**CCBP2**	0.68	±	0.59

35	**CCKAR**	1.26	±	0.31
36	**CCKBR**	1.94	±	0.32
37	**CCR1**	1.08	±	1
38	**CCR2**	0.85	±	0.27
39	**CCR3**	-1.07	±	0.37
40	**CCR4**	-0.18	±	0.2
41	**CCR5**	0.06	±	0.52
42	**CCR6**	-0.69	±	0.18
43	**CCR7**	-1.7	±	0.44
44	**CCR8**	-0.16	±	0.7
45	**CCR9**	-1.02	±	0.3
46	**CCR10**	-0.45	±	0.16
47	**CD97**	-0.59	±	0.57
48	**CMKLR1**	0.52	±	0.38
49	**CX3CR1**	0.02	±	0.67
50	**CXCR1**	-0.78	±	0.45
51	**CXCR2**	1.21	±	0.5
52	**CXCR3**	-2.94	±	0.33
53	**CXCR5**	-5	±	0.33
54	**CXCR6**	-0.75	±	0.1
55	**CysLTR1**	-0.8	±	0.26
56	**CysLTR2**	0.9	±	0.46
57	**D1R**	-2.94	±	0.14
58	**D2R**	-4.9	±	0.48
59	**D3R**	0.23	±	0.64
60	**D4R**	1.81	±	0.49
61	**D5R**	-1.61	±	0.23
62	**DARC**	-0.28	±	0.38
63	**DP**	-1.64	±	1.55
64	**DP2**	-2.95	±	1.46
65	**EBI2**	-0.27	±	0.46
66	**EP1**	-0.19	±	0.53
67	**EP2**	-1.42	±	0.39
68	**EP3**	0.59	±	0.25
69	**EP4**	1.07	±	0.37
70	**ETA**	0.05	±	0.38
71	**ETB**	-1.91	±	0.09

72	**FFA1**	-2.44	±	0.18
73	**FFA2**	-2.02	±	0.41
74	**FFA3**	-1.97	±	0.3
75	**FFA4**	-3.87	±	0.71
76	**FP**	2.62	±	0.94
77	**FPR1**	0.9	±	0.11
78	**FPR2**	3.19	±	0.34
79	**FPR3**	1.29	±	0.26
80	**FZD4**	1.4	±	0.33
81	**G2A**	0.49	±	0.35
82	**GABBR1**	-0.73	±	0.29
83	**GALR1**	0.02	±	0.43
84	**GALR3**	0.9	±	0.49
85	**GCGR**	0.99	±	0.55
86	**GHRHR**	0.15	±	0.18
87	**GHSR**	0.54	±	0.36
88	**GIPR**	-0.75	±	0.18
89	**GIT2**	0.67	±	0.13
90	**GLP2R**	0.69	±	0.37
91	**GNRHR**	3.08	±	0.93
92	**GPBA**	1.92	±	0.52
93	**GPER**	3.88	±	0.54
94	**GPR1**	-0.28	±	0.16
95	**GPR3**	-4.08	±	0.9
96	**GPR4**	-2.15	±	0.73
97	**GPR6**	-3.63	±	1.5
98	**GPR12**	-3.63	±	1.3
99	**GPR15**	-2.53	±	2
100	**GPR17**	0.9	±	0.29
101	**GPR18**	-0.91	±	1.17
102	**GPR20**	0.17	±	0.43
103	**GPR21**	0.62	±	0.23
104	**GPR22**	-0.86	±	0.22
105	**GPR25**	-2.29	±	0.3
106	**GPR26**	-6.45	±	0.37
107	**GPR27**	-0.81	±	0.21
108	**GPR31**	1.82	±	0.7

109	**GPR32**	0.48	±	0.55
110	**GPR33**	1.36	±	0.42
111	**GPR34**	0.35	±	0.56
112	**GPR35**	-4.24	±	0.51
113	**GPR37**	-6.47	±	0.57
114	**GPR38**	-0.84	±	0.13
115	**GPR39**	-3.47	±	2.27
116	**GPR45**	-0.66	±	0.54
117	**GPR52**	-2.23	±	2.27
118	**GPR55**	1.49	±	0.17
119	**Gpr56**	-0.9	±	0.35
120	**GPR61**	-1.87	±	1.56
121	**GPR64**	-0.8	±	0.33
122	**GPR65**	-3.94	±	1.36
123	**GPR68**	-0.34	±	0.06
124	**GPR75**	0.35	±	0.43
125	**GPR77**	1.17	±	0.79
126	**GPR78**	-4.95	±	0.68
127	**GPR81**	-1.18	±	0.31
128	**GPR82**	0.34	±	0.54
129	**GPR83**	-2.27	±	0.7
130	**GPR84**	1.87	±	0.21
131	**GPR85**	1.33	±	0.25
132	**GPR87**	0	±	1.24
133	**GPR88**	-0.14	±	0.4
134	**GPR101**	0.64	±	0.08
135	**GPR107**	-1.5	±	0.39
136	**GPR109A**	3.38	±	0.23
137	**GPR119**	4.5	±	0.63
138	**GPR133**	2.21	±	0.2
139	**GPR135**	2.27	±	0.18
140	**GPR139**	1.81	±	0.85
141	**GPR141**	1.19	±	0.33
142	**GPR142**	1.9	±	0.31
143	**GPR146**	2.5	±	0.21
144	**GPR148**	1.31	±	0.19
145	**GPR149**	-1.13	±	0.59

146	**GPR150**	-1.86	±	1.22
147	**GPR153**	-1	±	0.7
148	**GPR156**	0.06	±	0.34
149	**GPR157**	1.35	±	0.35
150	**GPR161**	0.96	±	0.4
151	**GPR173**	1.75	±	1.04
152	**GPR174**	1.02	±	0.48
153	**GPR182**	-2.65	±	0.44
154	**GPRC5A**	1.07	±	0.39
155	**GPRC5B**	0.56	±	0.49
156	**GPRC5D**	1.95	±	0.25
157	**GRM2**	2.01	±	0.21
158	**GRPR**	0.57	±	0.32
159	**H1**	0.73	±	0.44
160	**H2**	2.36	±	1.23
161	**H3**	2.05	±	0.23
162	**H4**	2.3	±	0.87
163	**HCRTR1**	-2.49	±	0.44
164	**HCRTR2**	-1.35	±	0.35
165	**IP**	-1.41	±	0.12
166	**KISS1R**	-2.95	±	0.8
167	**LGR5**	-3.09	±	0.69
168	**LGR6**	0.58	±	1.81
169	**LPA1**	-2.03	±	0.25
170	**LPA2**	-2.85	±	0.9
171	**LPA3**	0.73	±	0.44
172	**LPA4**	-0.28	±	0.95
173	**LPA5**	2.27	±	0.87
174	**LPA6**	-1.29	±	0.31
175	**M1**	-1.12	±	0.72
176	**M2**	-0.47	±	0.69
177	**M3**	-0.62	±	0.35
178	**M4**	-1.27	±	1.11
179	**M5**	-1.59	±	0.43
180	**MAS1**	-0.19	±	0.42
181	**MAS1L**	0.33	±	0.39
182	**MC1R**	-1.69	±	0.11

183	**MC2R**	-2.39	±	0.26
184	**MC3R**	-1.24	±	0.8
185	**MC4R**	-1.25	±	0.29
186	**MC5R**	-2.1	±	0.83
187	**MCHR1**	0.36	±	0.81
188	**MCHR2**	-0.75	±	0.99
189	**MLNR**	-1.42	±	1.25
190	**mMAS1**	-7.16	±	0.6
191	**Mrga1**	-0.64	±	0.41
192	**Mrga2a**	-4.71	±	0.37
193	**Mrga3**	-3	±	0.11
194	**Mrga4**	-2.07	±	0.63
195	**Mrga7**	-1.01	±	1.1
196	**Mrgb1**	-1.13	±	1.35
197	**Mrgb2**	-0.12	±	0.7
198	**Mrgb3**	-0.84	±	0.39
199	**Mrgb4**	-0.05	±	0.52
200	**Mrgb5**	-2.38	±	0.21
201	**Mrgb8**	-6.93	±	0.27
202	**Mrgb13**	-2.1	±	1.23
203	**MRGD**	-1.91	±	2.81
204	**Mrgd**	-12.08	±	0.46
205	**MRGE**	1.89	±	0.75
206	**Mrge**	-2.12	±	0.35
207	**MRGF**	3.01	±	0.39
208	**Mrgf**	-3.24	±	0.22
209	**MRGG**	2.27	±	0.17
210	**Mrgg**	-6.9	±	0.58
211	**Mrgh**	-4.5	±	0.48
212	**MRGX1**	2.36	±	0.34
213	**Mrgx1**	-7.34	±	0.49
214	**MRGX2**	0.21	±	0.06
215	**Mrgx2**	-3.38	±	0.07
216	**MRGX3**	2.66	±	1.22
217	**MRGX4**	0.14	±	1
218	**MT1R**	-2.71	±	0.3
219	**MT2R**	-0.32	±	1.03

220	**NK1**	-0.2	±	0.37
221	**NK2**	-0.93	±	0.44
222	**NK3**	1.12	±	0.44
223	**NMBR**	-1.77	±	0.26
224	**NMUR1**	1.15	±	0.23
225	**NMUR2**	3.76	±	1
226	**NPBWR1**	-1.51	±	0.24
227	**NPBWR2**	-1.42	±	0.75
228	**NPFFR1**	-1.1	±	0.24
229	**NPFFR2**	-0.67	±	0.82
230	**NPY1R**	-0.76	±	0.19
231	**NPY2R**	-0.66	±	0.24
232	**NPY5R**	-1.14	±	0.61
233	**NTSR1**	-1.67	±	0.18
234	**NTSR2**	-2.46	±	0.31
235	**OPRD1**	1.32	±	1.09
236	**OPRK1**	-1.33	±	0.7
237	**OPRL1**	-0.5	±	0.26
238	**OPRM1**	-1.98	±	0.45
239	**OXER1**	0.2	±	0.65
240	**OXGR1**	-0.2	±	0.12
241	**OXTR**	-0.18	±	1.04
242	**P2RY10**	-1.3	±	0.88
243	**P2Y1**	-0.23	±	1.21
244	**P2Y2**	-1.36	±	0.86
245	**P2Y4**	0.57	±	0.78
246	**P2Y6**	-1.39	±	1.04
247	**P2Y8**	3.44	±	0.91
248	**P2Y11**	-2.67	±	0.61
249	**P2Y12**	1.22	±	0.49
250	**P2Y13**	1.17	±	0.7
251	**P2Y14**	1.89	±	0.21
252	**PAC1**	2.36	±	0.29
253	**PAFR**	-0.56	±	0.57
254	**PAR1**	3.67	±	0.81
255	**PAR2**	1.26	±	0.27
256	**PAR3**	2.33	±	0.71

257	**PAR4**	0.69	±	0.86
258	**PRLHR**	1.5	±	0.04
259	**PROKR2**	2.27	±	0.72
260	**PTH1R**	1.29	±	0.9
261	**PTH2R**	2.09	±	0.21
262	**RGR**	4.53	±	0.23
263	**RXFP2**	3.39	±	0.53
264	**RXFP3**	2.65	±	0.52
265	**RXFP4**	0.78	±	0.26
266	**S1P1**	1.29	±	0.28
267	**S1P2**	0.02	±	0.35
268	**S1P3**	0.98	±	0.29
269	**S1P4**	-0.03	±	0.11
270	**S1P5**	1.05	±	0.18
271	**SSTR1**	1.92	±	0.67
272	**SSTR2**	2.85	±	0.4
273	**SSTR3**	2.44	±	1.81
274	**SSTR4**	1.47	±	0.4
275	**SSTR5**	-0.65	±	0.32
276	**SUCNR1**	1.33	±	0.34
277	**TAAR1**	2.28	±	0.76
278	**TAAR2**	2.22	±	0.28
279	**TAAR5**	1.1	±	0.69
280	**TAAR6**	2.25	±	0.44
281	**TAAR8**	1.17	±	0.39
282	**TAAR9**	0.88	±	0.38
283	**TAS2R1**	3.91	±	0.77
284	**TAS2R5**	0.1	±	0.23
285	**TAS2R7**	-0.18	±	0.21
286	**TAS2R8**	-0.67	±	0.1
287	**TAS2R9**	1.83	±	0.52
288	**TAS2R10**	4.17	±	0.37
289	**TAS2R13**	0.76	±	0.42
290	**TAS2R14**	1.26	±	0.17
291	**TAS2R19**	1.63	±	0.34
292	**TP**	0.39	±	0.32
293	**TRHR**	0.53	±	0.24

294	**TSHR**	0.71	±	0.44
295	**UTS2R**	-1.3	±	0.48
296	**V1A**	-0.29	±	0.32
297	**V1B**	0.47	±	0.39
298	**V1PR2**	0.16	±	0.37
299	**V2**	0.86	±	0.4
300	**VN1R1**	2.25	±	0.35
301	**XCR1**	6.37	±	0.59
302	**α1A**	2.89	±	0.53
303	**α1B**	5.74	±	1.21
304	**α1D**	4.49	±	0.42
305	**α2A**	6.52	±	0.33
306	**α2B**	3	±	0.75
307	**α2C**	4.31	±	0.17
308	**β1**	5.02	±	0.86
309	**β2**	9.09	±	0.17
310	**β3**	6.54	±	0.51
